# Improved solar still productivity using PCM and nano- PCM composites integerated energy storage

**DOI:** 10.1038/s41598-024-65418-1

**Published:** 2024-07-06

**Authors:** G. Murali, P. Ramani, M Murugan, P. V. Elumalai, Nayani Uday Ranjan Goud, S. Prabhakar

**Affiliations:** 1 Department of Mechanical Engineering, Koneru Lakshmaiah Education Foundation, Green Fields, Vaddeswaram, Guntur, Andhra Pradesh 522502 India; 2 Department of Electronics and Communication Engineering, SRM Institute of Science and Technology, Ramapuram, Chennai, Tamilnadu 600089 India; 3 Department of Mechanical Engineering, Vivekanandha College of Engineering for Women, Tiruchengode, Namakkal, Tamilnadu 637205 India; 4 Department of Mechanical Engineering, Aditya University, Surampalam, Andhra Pradesh 533437 India; 5 Department of Aeronautical Engineering, MLR Institute of Technology, Hyderabad, Telangana India; 6 Department of Mechanical Engineering, Wollo University, Dessie, Ethiopia; 7 Department of Mechanical Engineering, Saveetha School of Engineering, Saveetha Institute of Medical and Technical Sciences (SIMATS), Chennai, Tamil Nadu 60210 India; 8 Research Fellow, Faculty of Engineering. Shinawatra University, Bang Toei, 12160 Thailand

**Keywords:** Al2O3, Nano-PCM, Energy storage, PCM, Productivity, Efficiency’, Chemistry, Energy science and technology, Engineering

## Abstract

The study investigates the impact of Phase Change Material (PCM) and nano Phase Change Materials (NPCM) on solar still performance. PCM and a blend of NPCM are placed within 12 copper tubes submerged in 1 mm of water to enhance productivity. Thermal performance is assessed across four major scenarios with a fixed water level of 1 mm in the basin. These scenarios include the conventional still, equipped with 12 empty copper rods and 142 g of PCM in each tube, as well as stills with NPCM Samples 1 and 2. Sample 1 contains 0.75% nanoparticle concentration plus 142 g of PCM in the first 6 tubes, while Sample 2 features 2% nanoparticle concentration plus 142 g of PCM in the subsequent 6 tubes. Aluminum oxide (Al2O3) nanoparticles ranging in size from 20 to 30 nm are utilized, with paraffin wax (PW) serving as the latent heat storage (LHS) medium due to its 62 °C melting temperature. The experiments are conducted under the local weather conditions of Vaddeswaram, Vijayawada, India (Latitude-80.6480 °E, Longitude-16.5062 °N). A differential scanning calorimeter (DSC) is utilized to examine the thermal properties, including the melting point and latent heat fusion, of the NPCM compositions. Results demonstrate that the addition of nanoparticles enhances both the specific heat capacity and latent heat of fusion (LHF) in PCM through several mechanisms, including facilitating nucleation, improving energy absorption during phase change, and modifying crystallization behavior within the phase change material. Productivity and efficiency measurements reveal significant improvements: case 1 achieves 2.66 units of daily production and 46.23% efficiency, while cases 2, 3, and 4 yield 3.17, 3.58, and 4.27 units of daily production, respectively. Notably, the utilization of NPCM results in a 60.37% increase overall productivity and a 68.29% improvement in overall efficiency.

## Introduction

In various regions of the world, pure water is becoming increasingly precious and in short supply. Only a small amount of the water that exists on earth is freshwater. The majority of it is buried or trapped in ice caps and glaciers, making it inaccessible. Just 0.3% of the scarce freshwater available is surface water that is easily accessible. The shortage of fresh water in a region is determined by its location. Instead, desalination, a cutting-edge process, has the capacity to produce millions of liters of pure distilled water each day. Smaller household applications in rural and village settings that don’t contain chemicals or release greenhouse gases can employ the solar still with the technology. Through a feeder pipe, impure water is introduced into the still’s basin, where it slowly evaporates and is evenly distributed on the transparent greenhouse glass cover. Instead of falling as rain, the input water condenses along the glass until it reaches the gutter, where it flows into a collection vessel^1–3^.

Within the specially constructed glass plate enclosure, solar radiation heats the input water, inducing a phase change and leading to vapor condensation. This process effectively prevents the growth of bacteria and pathogens, mitigating the risk of waterborne diseases. As the water evaporates, anything heavier than water, including microorganisms and heavy metals, is left behind and collects as sludge at the bottom. Lighter hydrocarbons, such as acetone or gasoline, rise alongside the water but can be easily discarded by eliminating the initial drops from the still. The resulting distilled water droplets then flow into a pure water outlet at the base of the unit. However, a notable drawback of solar desalination is its lower productivity. To address this limitation^4,5^, various enhancements have been developed for the solar still. These include the utilization of phase change materials (PCMs), sponge cubes, sand, and other absorbent components to augment production. Among these materials, PCM stands out as the most accurate and efficient in storing heat. PCM, employed as both sensible and latent heat storage (LHS) media, has proven to be non-corrosive and making it a reliable medium for high-temperature heat storage^6,7^.

When selecting a phase change material (PCM), it is important to consider various factors such as its melting point, density, durability, and other properties. PCM selection should be based on specific application requirements, such as the desired operating temperature range, the amount of thermal energy to be stored and released, and the essential lifetime of the material. High melting point PCMs, such as paraffin wax (PW), fatty acids, and salt hydrates, are commonly used in various heating and cooling applications because they can store and release a large amount of thermal energy within a narrow temperature range. PW is another PCM that has a high melting point range between 40 and 90 °C, making it suitable for use in many heating and electrical applications^8–10^. Hydrated salts are a type of PCM that can be used for energy storage. They are typically composed of a salt that has water molecules trapped within its crystal lattice structure. When the temperature rises, the water molecules within the salt crystal structure melt, absorbing heat and storing thermal energy. Hydrated salts have several advantages as PCMs. As mentioned, they have a high latent heat of fusion per weight^11–13^.

The review offers a thorough and comprehensive examination of recent studies focusing on the integration of PCMs in various low-temperature applications, including building envelopes, passive systems within buildings, solar collectors, solar photovoltaic systems, and solar desalination systems. Additionally, it discusses techniques aimed at enhancing heat transfer in PCM systems. Across all application studies, it is consistently observed that the performance of these applications improves upon the application of PCM technology^14^. Research has shown that using phase change material (PCM) can increase the solar efficiency of desalination systems by up to 40%. In one study, researchers employed a parabolic trough collector and a shell-and-tube heat exchanger to generate steam for desalination. By incorporating PCM into the heat exchanger, they achieved a thermal efficiency of 42%, compared to 30% without PCM^15^. The single slope solar still was the subject of experimental research using PCM combined with hollow cylindrical pin fins. The conventional still was modified by adding PCM and fins to improve its heat transmission capabilities and productivity at night. According to observations, a still with surrounded PCM pin fins has greater productivity and a 7-h storage capacity compared to a still with a single PCM, which has a 5-h storage capacity^16^.

Investigators found that employing a composite phase change material (CPCM) yields the best results compared to using a phase change material (PCM). They conducted an experimental, comparative, and economic study on solar stills to boost their storage capacity. When compared to a straightforward solar still, the productivity of the CPCM made up of the organic phase change material R48 and the inorganic phase change material capric palmitic acid increased by 92%^17^. The experiment involved filling copper cylinders with a combination of three distinct PCMs—paraffin wax (PW), stearic acid, and lauric acid—and setting them in a still basin at evenly spaced points with variable water depths. During the experiment, it was discovered that utilizing paraffin wax (PW), stearic acid, and lauric acid, in that order, increased distillate production by 1120, 1050, 950 ml/m^2^/day^18^. The use of a 4-kg PCM in solar still resulted in distillate productivity 18.6% and 27.7% higher than that achieved with 2- and 6-kg PCM, respectively. This improvement can be attributed to the incomplete charging of PCM at 6 kg and the underutilization of total daytime available energy with 2-kg PCM. Furthermore, compared to conventional solar still, distillate productivity increased by 30%^19^. It was reported that compared to traditional solar still setups, the paraffin wax-based solar still, achieving a distillate yield of 3.96 kg/m^2^, enhances productivity by 28.14%. Additionally, introducing floating wicks inside the basin of PCM-solar still led to a further increase in distillate productivity by 32%^20^. The performance of various solar still designs integrated with PCM. Additionally, it examined the impact of nanoparticles on PCM-integrated solar stills with different absorber designs and configurations. Compared to conventional solar stills, the heat transfer techniques in PCM-based solar stills can substantially enhance overall distillate productivity: Tubular solar still by 218%, followed by single basin single slope solar still at 149%, pyramidal at 125%, respectively. Moreover, nighttime productivity increased by 235%^21^. An experimental setup was used, employing composite phase change material (CPCM), to carry out numerical modeling of a solar still. Erythritol and expanded graphite (EG), with a melting temperature of 119 °C, constitute the CPCM. Familiar as an excellent material for heat storage, this CPCM, containing 3 weight percent expanded graphite, is placed into aluminum pipes within evacuated tubes. Heat pipes are then submerged in the phase change material (PCM) to enhance heat transfer between the pipes. It was found that the storage efficiency had improved to 40.7% after all observations were complete^22–24^. The process is based on nanofluid structures, with PCM integrated into the internal heat transfer mechanism. It has been noted that employing an enhancement strategy for PCM nanofluids alongside double slope solar still leads to a notable increase in the production of double slope solar still distillate^25^.

Nanotechnology is a field of science and engineering that deals with the study, manipulation, and design of materials at the nanoscale level. The use of nanotechnology in the field of thermal energy storage (TES) has shown promising results in overcoming the limitations of phase change materials (PCM). One approach is to incorporate nano-sized particles into the PCM matrix to enhance its thermal conductivity. These particles can be made of materials such as graphene, carbon nanotubes, or metal oxides, and their high surface area-to-volume ratio can improve heat transfer within the PCM. By increasing the thermal conductivity of the PCM, the heat transfer rate can be improved, and the overall thermal performance of the TES system can be enhanced^26^. Mixing a high-thermal conductivity graphite plate with paraffin wax resulted in a substantial improvement in the total productivity of the solar still. Specifically, there was a 62% increase during daytime hours and a remarkable 235% increase during night-time hours^27^. Three solar stills, all with identical dimensions, were designed and fabricated: one utilizing PCM, another integrated with nano phase change material (NPCM) composed of paraffin and 0.5% mass of silica nanoparticles and the third being a conventional setup. Experimental results demonstrated that incorporating PCM and NPCM increased fresh water production by 51.22% and 67.07%, respectively^28^.

Researchers have been drawn to nanotechnology to the point where they have started testing various nanoparticles on solar stills. To investigate the interaction between Phase Change Material (PCM), flake graphite nanoparticles, and film cooling, S.W. Shamshir et al. carried out an experiment. The experiment was conducted in four different scenarios by the author: 1. Adding 0.5% flake nanoparticles of graphite to water 2. Flake nanoparticles with PCM 3. Nanoparticles that flake with film cooling 4. Mentioned above the three situations. In case 4, the results were more favorable than in case 2, case 1, or case 3^29^. Sarparaju.et.al utilized a 1 mm-thick copper sheet to fabricate an absorber basin measuring 100 cm × 60 cm × 5 cm (0.6 m^2^ absorber area)^30^. In another study Hyder et al.^31^ the inner basin dimensions were 100 cm × 100 cm, with varying heights of 528 mm (higher side) and 60 mm (lower side). Their still incorporated a condensing surface made of a 4 mm-thick window-type glass^31^. In comparison, our solar still measures 1060 mm in length and 1774 mm in width, with a height ranging from 135 mm at the front to 404 mm at the back.

According to the literature, it has been observed that the efficiency and productivity of solar stills increase when the thermal conductivity of phase change material (PCM) is enhanced through the addition of nanoparticles. In this present study, a comparison between a conventional solar still and a modified version is conducted, experimenting with different ratios of nano-phase change material (NPCM) to identify the most effective one. This could be done by highlighting how variations in NPCM ratios can significantly impact the thermal conductivity, heat storage capacity, and overall performance of solar still systems. By systematically comparing different NPCM ratios, the study aims to identify the most effective composition for enhancing the efficiency and productivity of solar stills.

## Experimental analysis

**Figure 1:**
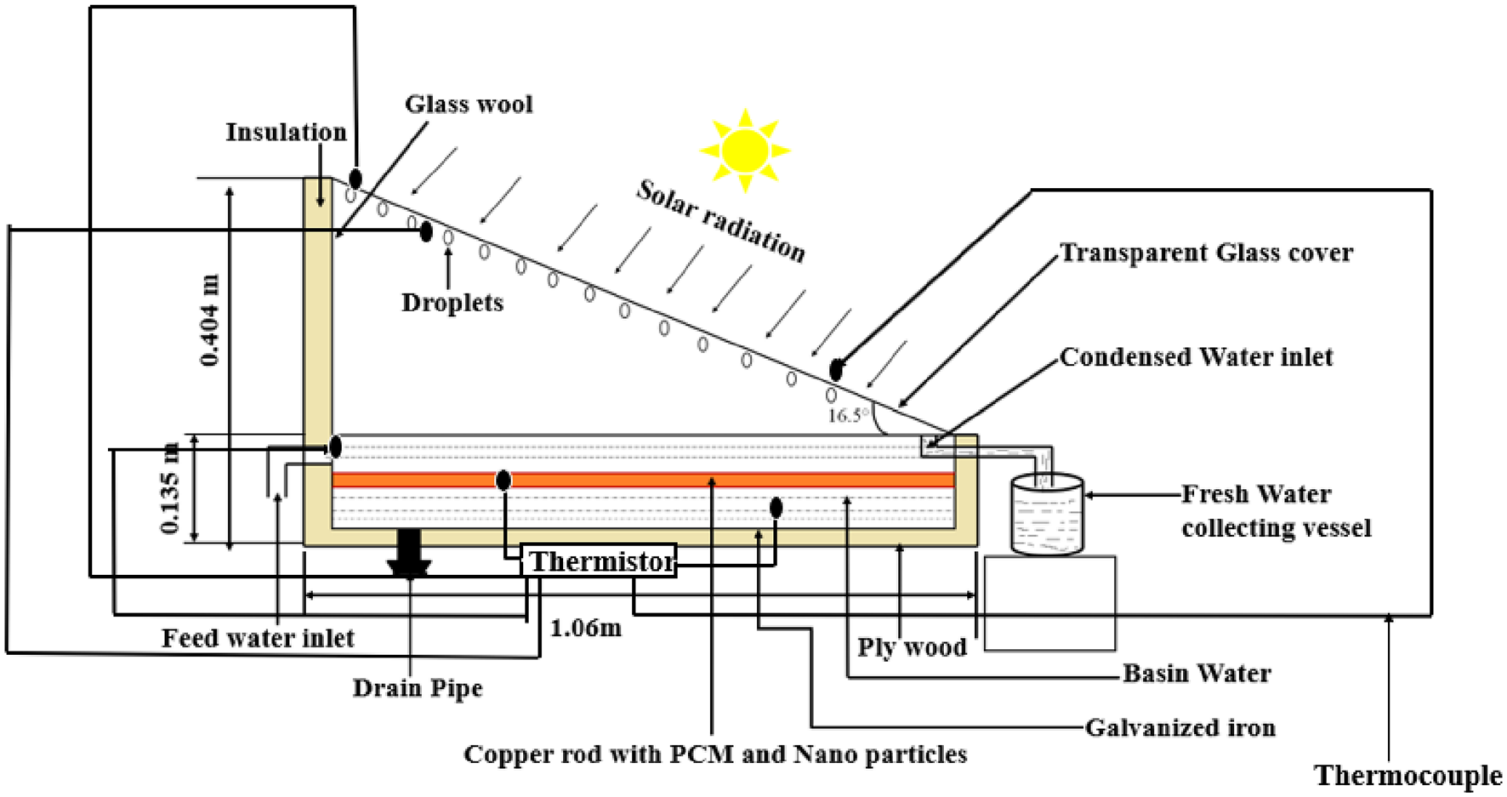
Schematic diagram of experimental setup.

**Figure 2:**
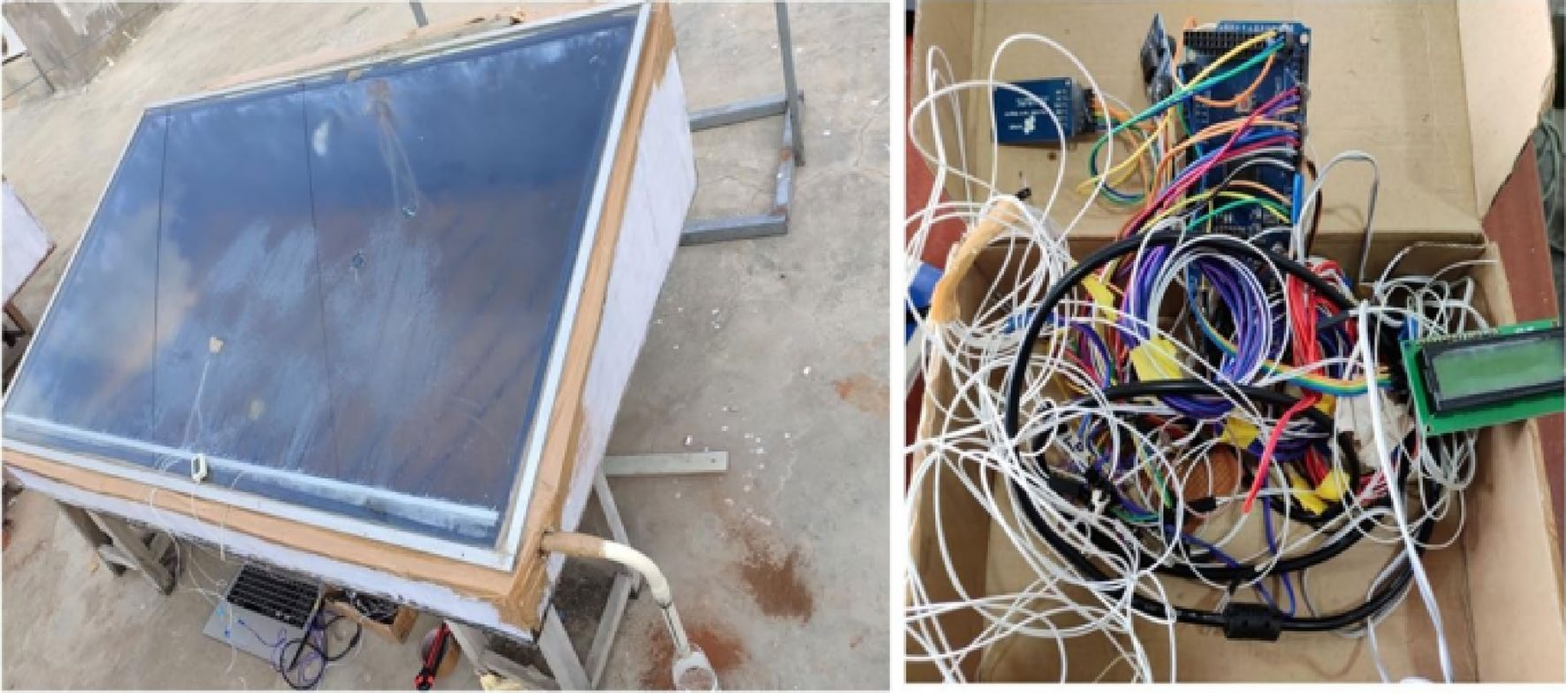
Arudino linked to a 100 k thermistor and a solar still are part of an experimental setup on the bottom.

The experiment was carried out on a solar still with a single slope that was developed. Iron that’s been galvanized makes up the still basin. With a 3 mm thickness, glass wool and thermocol are employed as insulation. A transparent glass cover with a 5 mm thickness is angled 16.5° on the upper side of the still collect moisture. The selection of the tilt angle of 16.5° may have been based on factors such as the latitude of the location (16.5062 °N) and the angle of incidence of solar radiation. In regions near the equator, such as Vaddeswaram, Vijayawada, a tilt angle close to the latitude angle can maximize solar radiation capture throughout the year. The still is rectangle in design, measuring 1060 mm long by 1774 mm wide. Its height is 404 mm at the back and 135 mm at front side. The still basin is painted black to enhance the absorptivity of solar radiation. Twelve copper rods, each with a diameter of 15 mm, filled with phase change material (PCM) and nano phase change material (NPCM), are immersed in water and placed at equidistant positions. The use of 12 rods in the solar still setup serves multiple purposes, including optimizing latent heat storage capacity and enhancing productivity. These rods are strategically positioned to ensure the even distribution of heat absorption across the water’s surface, leading to uniform heating and improved evaporation rates. Additionally, the 12 rods significantly increase the total surface area available for heat absorption, allowing for greater interaction with solar radiation and enhanced heat transfer to the water. By filling each rod with PCM or NPCM, the setup maximizes latent heat storage capacity, as these materials can store large amounts of thermal energy during phase change. Ultimately, the utilization of 12 rods enhances the efficiency of the solar still by capturing more solar energy and converting it into thermal energy, resulting in higher productivity and efficiency of the system. The experimentation utilized four distinct situations, and the water level in the basin was 1 mm: Case 1 is a standard still, Case 2 has 12 empty rods, Case 3 contains 12 rods with 142 g of PCM in each tube, and Case 4 contains 12 rods with contain 0.75% NPCM in 6 rods and 2% NPCM in the remaining 6 rods. The schematic design and modified experimental setup of a solar still linked to an Arduino are shown in Figs. 1 and 2, respectively. Table 1 lists the specifications of the materials used in the still.

**Table 1:** The specifications of the experimental setup.

Components	Utilized materials	Dimensions/quantity
Basin	Galvanized iron	163.4 × 90 mm
Coating	Black paint (Thermal sprayer)	2
Frame	Wood	–
Glass cover	Transparent glass	5 mm thickness
Insulation	Glass wool &Thermocol	7 mm each side
Nano particles	Aluminum oxide	25 g
PCM	Paraffin wax	1.993 kg
Storage pipe	Copper (12)	Diameter-15 mm; Length-87 mm; Thickness-0.2 mm

### Materials

The latent heat storage substance is solid paraffin with a melting temperature of 62 °C, which was purchased at chittinagar, Vijayawada, India. Al_2_O_3_ nanoparticles weighing 25 g were bought from GK Life Sciences in Guntur, India. The chosen melting point of the PCM solid paraffin at 62 °C is deemed suitable for our solar still system based on its compatibility with the anticipated operating temperature range. This temperature aligns well with the expected thermal conditions encountered during solar still operation, ensuring that the PCM undergoes phase change effectively within this range. Consequently, this facilitates efficient energy storage and release processes, thereby enhancing the overall performance and productivity of our solar still setup. Table 2 lists the characteristics of phase change material and nano phase change material.

**Table 2:** PCM and nanoparticles’ physical characteristics.

S.no	Property	PCM(Paraffin)	Nanoparticles (Al2O3)
1	Melting point	58–60 °C	–
2	Density	930 kg/m^3^	3.5–3.9 g/cm^3^
3	Thermal conductivity of the material	0.240 W/m K	35 W/m K
4	Latent heat	351.7 kJ/kg	–
5	Specific heat capacity of the material	2.14 kJ/kg K	880 J/kg K
6	Appearance	White	White

### Synthesis and sample preparation

**Figure 3:**
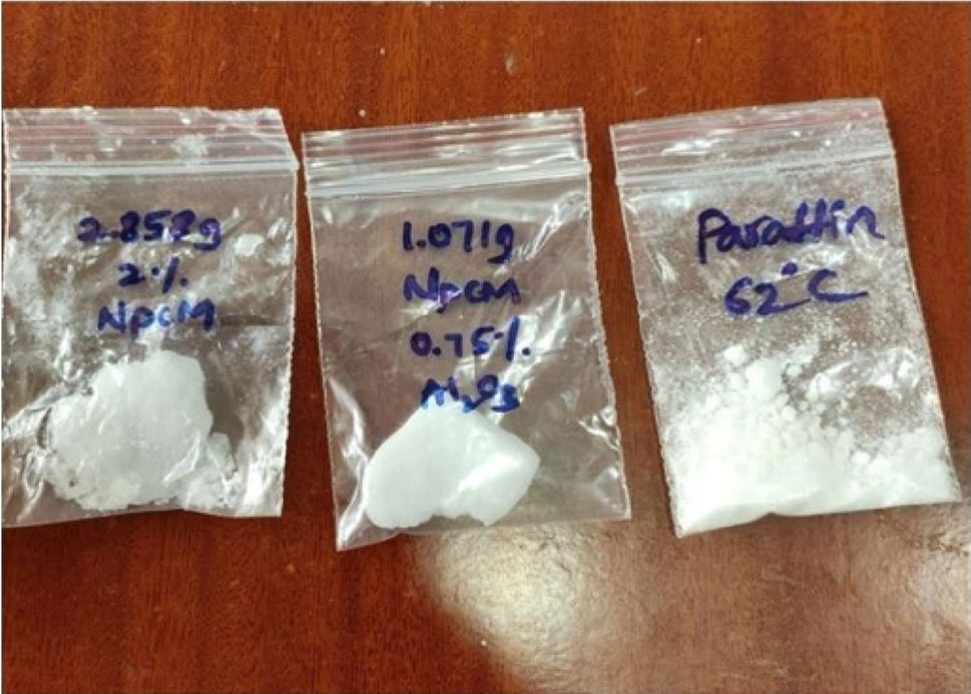
NPCM samples with 0.75% and 2% Al2O3.

**Figure 4:**
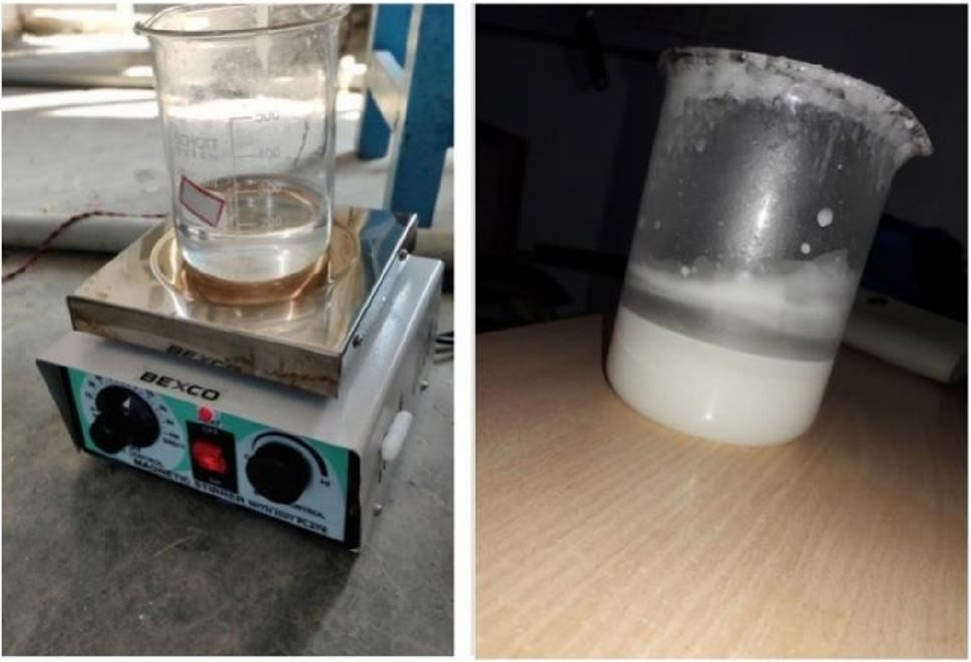
Thick NPCM solution on the right, and pure PCM with a magnetic stirrer on the left.

Nano phase change material (NPCM) refers to a type of phase change material (PCM) that contains nanoparticles, which enhances its thermal properties. As for mixing nano-PCMs, there are two common methods: the one-step approach and the two-step procedure. The one-step approach involves directly dispersing the nanoparticles into the PCM during the synthesis process. This method is relatively simple and requires fewer processing steps, but the distribution of nanoparticles may not be uniform, which can affect the overall thermal performance of the mixture. On the other hand, the two-step procedure involves adding the nanoparticles to the PCM after the synthesis process. This method allows for better control over the distribution of nanoparticles and can result in a more uniform mixture. Additionally, this method has been observed to be the most efficient way to assess the mixture’s thermal characteristics^32–34^. Solid paraffin wax (PW) was heated to a temperature above its melting point before mixing with the nanoparticles. For each tube, only 142 ml of paraffin are needed. Aluminum oxide (Al2O3) nanoparticles are introduced to liquid paraffin at concentrations of 0.75 and 2 percent as shown in Fig. 3, while maintaining the paraffin at a temperature above its melting point. To prevent the agglomeration of nanoparticles, the mixture of PCM and nanoparticles was agitated using a magnetic stirrer at 350 rpm for 45 min as shown in Fig. 4.

### Characterization and nano mixed PCM stabilization

Research experiment that involves manufacturing nano-blends with phase change material (PCM) and analyzing their thermal properties using different techniques such as differential scanning calorimeter (DSC), x-ray powder diffraction (XRD), and transmission electron microscopy (TEM). DSC is a common technique used to study the thermal behavior of materials, including their specific heat capacity, phase transition temperatures, and enthalpy changes. By analyzing the DSC curve, one can obtain information about the thermal stability and energy storage/release capacity of the PCM-nano blend^35^. XRD is a technique that can provide information about the crystal structure and phase composition of the material. It is useful in determining the size and distribution of the nanoparticles, as well as any changes in crystal structure due to the addition of PCM. TEM is a microscopy technique that allows visualization of the nanostructures, providing information about their size, shape, and morphology. It is particularly useful in studying the dispersion and agglomeration of the nanoparticles within the PCM matrix. Overall, the combination of DSC, XRD, and TEM analysis can provide a comprehensive understanding of the thermal properties and structure of the PCM-nano blends, which is essential for optimizing their performance in various applications^36–38^.

Differential Scanning Calorimetry (DSC) and Thermogravimetric Analysis (TGA) are fundamental techniques for assessing the thermal stability of novel phase change materials (NPCMs). In DSC analysis, a thermally stable NPCM typically exhibits consistent melting and solidification peaks across multiple heating and cooling cycles, indicating its ability to maintain phase transitions. Similarly, in TGA analysis, minimal weight loss in a thermally stable NPCM suggests little to no decomposition even after repeated heating cycles. While specific DSC and TGA curves at various thermal cycles would offer concrete evidence, their absence doesn’t diminish the importance of these techniques in evaluating NPCMs. Researchers commonly rely on DSC and TGA to gauge material performance in real-world conditions. Thus, the presence of consistent phase transitions in DSC curves and minimal weight loss in TGA curves are key indicators of thermal stability sought after in NPCM development.

### Data collection

In this experiment, we utilized a device called the Arduino Mega Max to record temperatures. NTC thermistors, capable of withstanding temperatures up to 150 °C, were used as the temperature sensors. In Fig. 1, the thermistor placed at the bottom of the solar still was used for temperature measurement. The six thermocouples are located at different positions, including the top and bottom of the glass cover, the center of the copper rod, the ambient environment, the evaporation surface, and within the basin water. Temperature measurements are taken at these locations every 30 min.

## Error and uncertainty analysis

Uncertainty analysis involves estimating the level of uncertainty associated with a particular measurement or data point. This uncertainty arises from a variety of sources, including measurement errors, variability in the sample, and limitations in the measurement equipment. By characterizing and quantifying this uncertainty, we can better understand the reliability and validity of our results, and make more informed decisions based on the data.

During sunny days from 9:00 am to 5:00 pm in the months of January through March, experiments were carried out. Temperature data is not collected after 5:00 pm because the primary heat source, solar radiation, diminishes. The PCM absorbs excess heat during the day, preventing rapid dissipation, and gradually releases stored thermal energy during cooler nighttime hours. This extends the solar still’s operation, enhancing efficiency, particularly in regions with prolonged sunlight and cooler nights. Therefore, collecting temperature data after 5:00 pm wouldn’t provide relevant insights into the system’s performance driven by solar energy. The temperatures of the copper rod, outside air, glass, evaporation, and basin water were measured using the NTC thermistor with a range of 150 °C. With the use of a measuring beaker, the distillate output is calculated. A solar power meter is used to detect solar radiation. Table 3 shows results of an investigation of the measurement instruments’ level of uncertainty.

**Table 3:** Error study of various measurement devices used in experiments.

S.no	Instrument used	Parameter	Symbol	Accuracy	Range	Least count	Uncertainty
1	Thermistor	Temperature	T	± 0.1 °C	150 °C	0.1 °C	± 0.1 °C
2	Solar power meter	Solar intensity	I(t)	± 10 w/m^2^	2000 w/m^2^	1 w/m^2^	± 1 w/m^2^
3	Measuring Beaker	Distillate output	M	± 0.01L	1 L	0.01 L	± 0.01 L
4	Measuring tape	Solar still length, width, height	L,W,H	± 12 cm	10 M	0.1 cm	± 0.1 cm

The efficiency of the solar still is calculated for all considered cases. The daily energy efficiency of the solar still is estimated using the following formula^39^.

$$\eta \, = \, (\sum P_{d} \times L) \, /A_{p} \times I_{d}$$

where P_d_ represents the total daily freshwater productivity in kilograms. *I*_*d*_ stands for the overall daily incident solar energy on the solar still, measured in joules per square meter (J/m^2^), *A*_*p*_ denotes the projected area of the solar still in square meters (m^2^), *Lav* is the latent heat of evaporation of water, measured in joules per kilogram (J/kg).

The productivity of the solar still defined as the difference between the productivity of modified distiller and conventional distiller divided by the productivity of the conventional distiller^31^.

$$P = (P_{d} )_{m} (P_{d} )_{c} /(P_{d} )_{c}$$

## Results and discussion

Throughout the experiment, many parameters including water temperature, basin temperature, glass temperature, evaporation temperature, and temperature of the rod were tracked hourly with a certain depth of water in basin 1 mm. However, it’s important to note that due to constraints, such as limited time and resources, our data collection was restricted to a single day. While we recognize the value of extending the study to include data over multiple days, the presented results provide insights into the system’s performance under specific experimental conditions.

### Radiation and the impact of different temperatures for different solar stills

**Figure 5:**
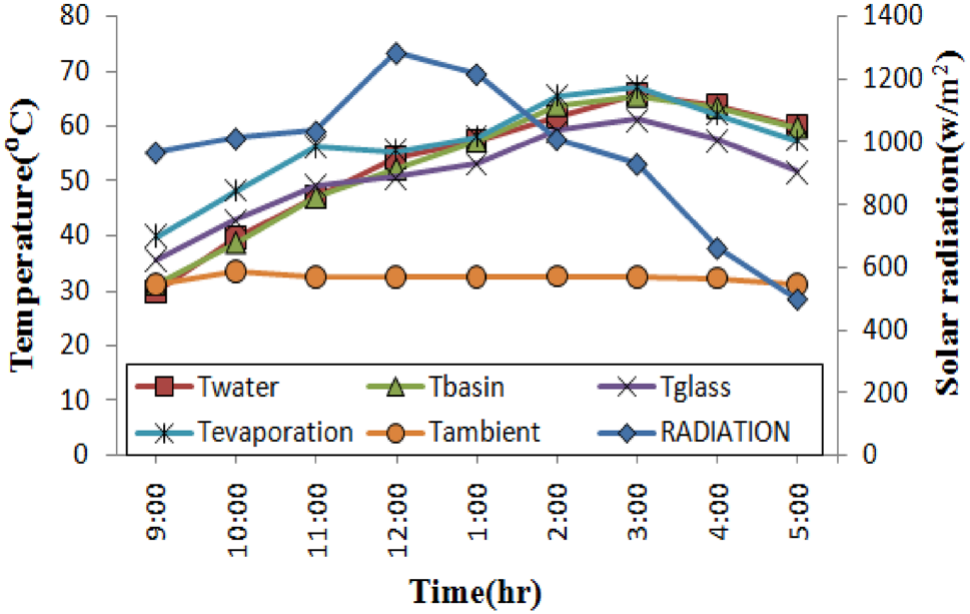
Temperature profiles over time for a conventional solar still.

**Figure 6:**
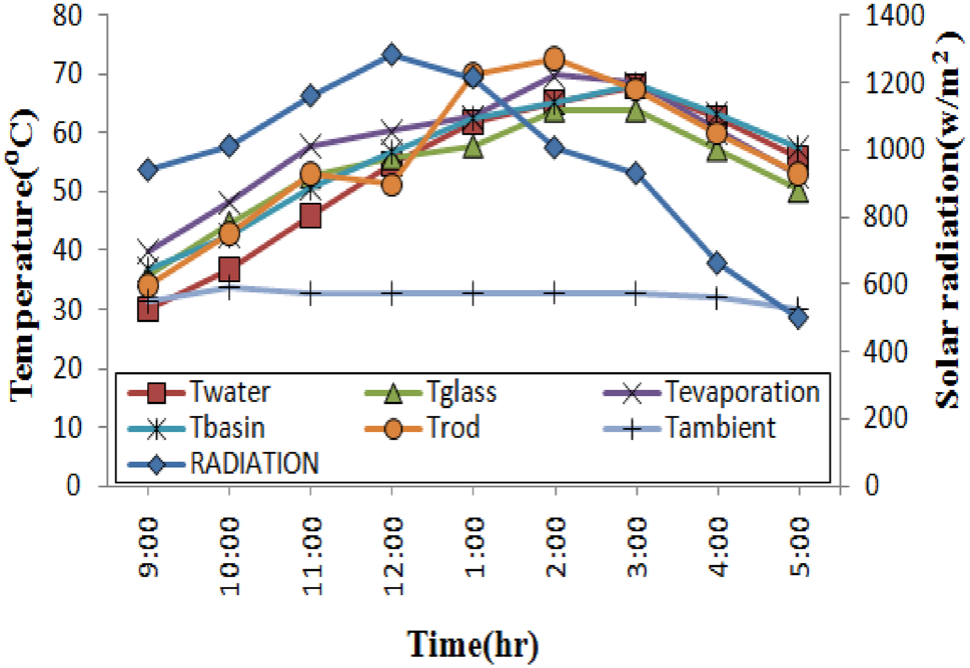
Temperature profiles over time for still with empty copper rods.

**Figure 7:**
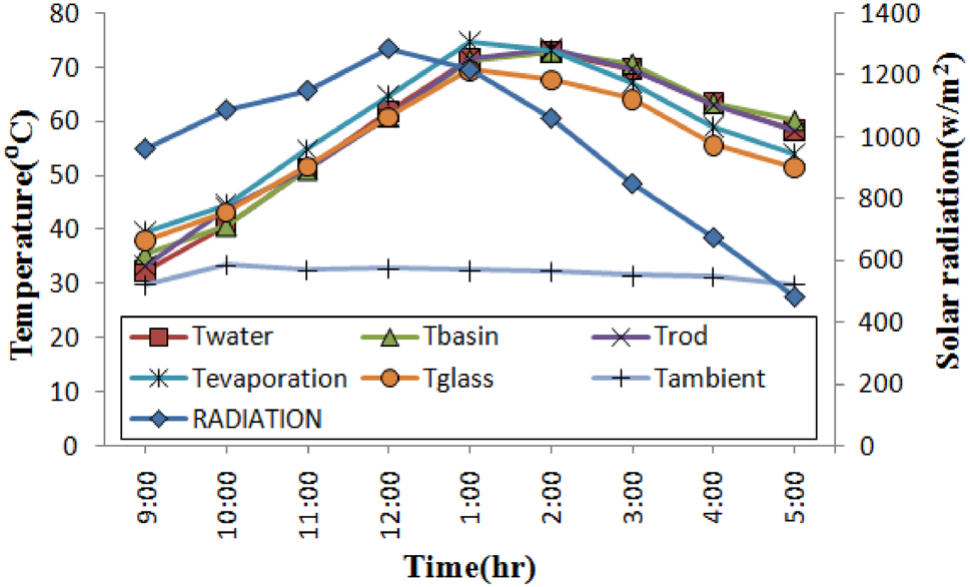
Temperature profiles over time for still with PCM rods.

**Figure 8:**
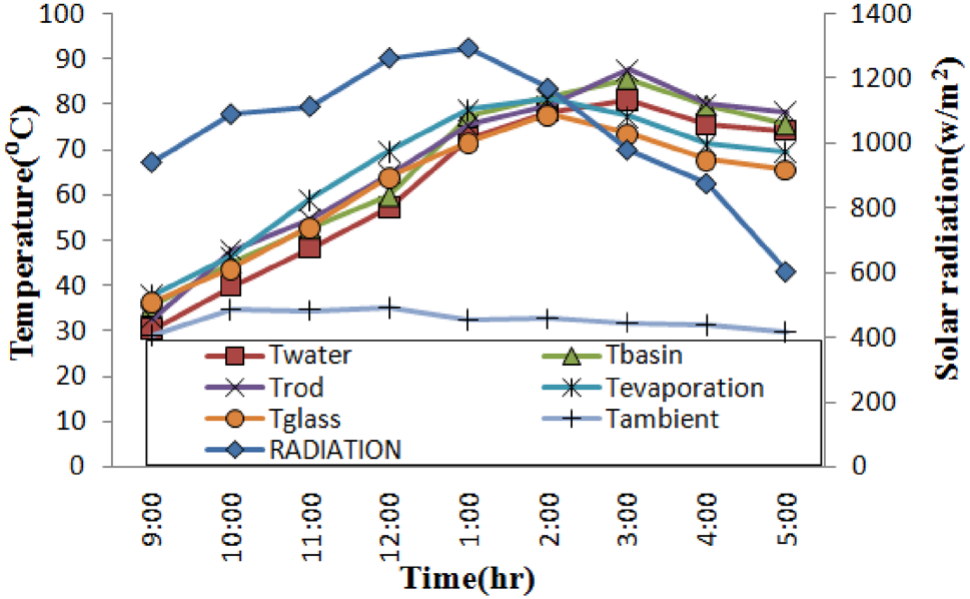
Temperature profiles over time for still with NPCM rod.

Solar radiation has an intensity range of 575–1286 W/m^2^. With the same solar intensities, the experiment was run on all 4 distinct stills. During the experiment at a fixed depth of water 1 cm and under the same climatic conditions, the thermal performance of stills with nano phase change material (NPCM) and phase change material (PCM) rods was compared to stills with empty rods and conventional stills. Figures 5, 6, 7 and 8 depicts the fluctuations in the temperatures of four distinct stills’ water, basin, glass, and rods as well as the ambient and evaporation temperatures and sun radiation. It was found that the amount of solar radiation steadily builds up until it reaches its greatest value at noon and then gradually drops until it reaches its lowest value at 5:00 pm. The maximum temperature for a conventional still was 61.2 °C for the glass, 67.1 °C for the evaporation, and 65.3 °C for the basin, as shown in Fig. 5. According to the Fig. 6, a still with empty rods had an extreme temperature of the water around 67.9 °C, whereas the extreme temperature for glass, evaporation, basin was 63.9 °C, 68.6 °C, and 68.3 °C.

In Fig. 7, the extreme temperature of the water is approximately 72.90 °C, while the extreme temperatures for the glass, evaporation, and basin for a still using phase change material (PCM) rods were 67.80 °C, 74.20 °C, and 72.80 °C, respectively. When using a still with nano phase change material (NPCM) rods, the maximum temperatures for the glass, evaporation, and basin were, respectively, 77.80 °C, 81.20 °C, and 85.70 °C as shown in Fig. 8. Empty rod, PCM rod, and NPCM rod had maximum temperatures of 72.60 °C, 73.50 °C, and 87.70 °C, respectively. The PCM used in the stills was similar and utilized above its melting point to provide sufficient energy storage during the charging time. By noon during the day, PCM in the stills begins to melt, and there is a significant variation among the water temperature in the basin and the temperature of the glass.

**Figure 9:**
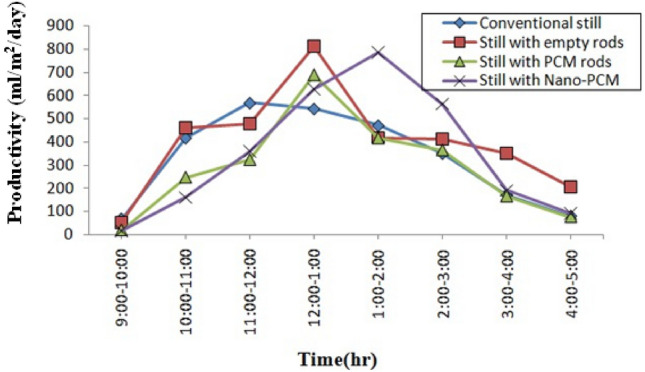
Hourly output of every type of still.

**Figure 10:**
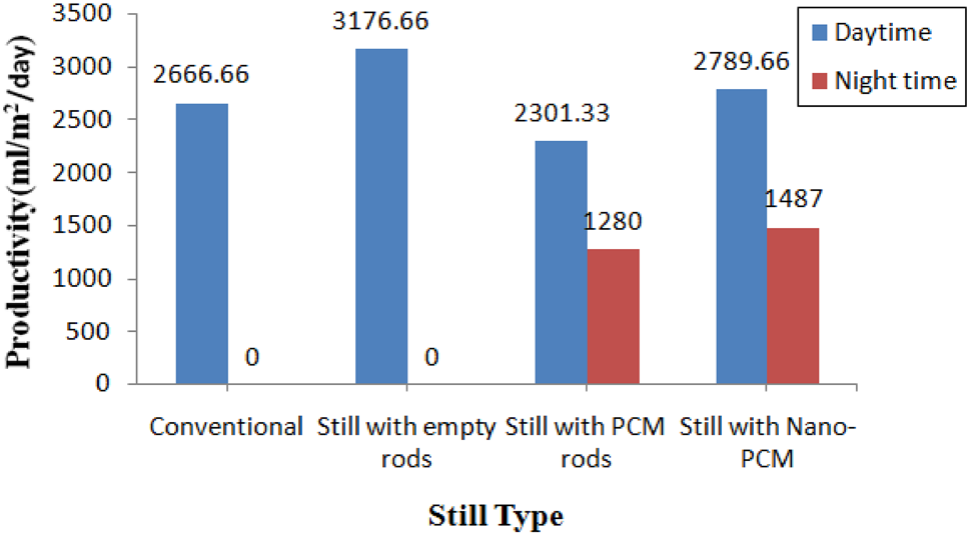
The output of different solar stills during the day and at night time.

The production of four stills per hour from 9:00 am to 5:00 pm is shown in Fig. 9, along with the output of stills with phase change material (PCM) and nano phase change material (NPCM) rods at night time as shown in Fig. 10. The productivity of the solar still depends on the amount of heat absorbed from solar radiation, peaking at 11:30 am and gradually declining after 3:00 pm. Consequently, still productivity increases until 1:00 pm, decreases thereafter, and no yield is produced from stills 1 and 2 after 5:00 pm.

The conventional still yields less output compared to the still with empty copper rods due to the inclusion of copper rods, which enhance radiation absorption from 9:00 am to 5:00 pm. Distilled water production is higher with empty copper rods because copper rods can absorb more radiation from the sun compared to phase change material (PCM) and nano phase change material (NPCM) rods. However, PCM and NPCM rods have their own special heat properties that affect water production during both the day and night. The NPCM still demonstrates higher productivity both during the day and at night time compared to the PCM still, attributed to the addition of nanoparticles. These nanoparticles enhance PCM’s thermal characteristics, thereby increasing hourly productivity. Consequently, the heat energy stored in PCM contributes during the discharge period from 5:00 pm to 9:00 am. Conventional and empty rod stills are productive only in the presence of radiation, while PCM and NPCM stills provide productivity throughout both day and night.

With the presence of latent heat energy in PCM, it helps to prolong the discharge time. During the melting of NPCM, the water temperature is maintained at a very high rate compared with PCM still water temperature. The temperature difference between water and glass is greater during the daytime and decreases at nighttime. Latent heat energy storage allows water to evaporate to a certain extent and stores the remaining energy to increase evaporation during nighttime discharge. The daytime productivity of conventional stills and stills with empty rods is 2.66 and 3.17 daily production, respectively, which is higher than that of PCM still and NPCM still with 2.30 and 2.78 daily production, respectively. Although the overall productivity of NPCM still is higher than that of other stills, the daytime productivity is lower than that of the other three stills. The productivity per day of NPCM still is higher at nighttime than that of other stills.

A still with an empty rod, a phase change material (PCM) still, and a nano phase change (NPCM) still each have a total (day time plus night time productivity) of 3.17, 3.58, and 4.27 daily production, respectively. The NPCM still has a 19.4% better nighttime production than the PCM still.

The overall efficiency of a solar still can be expressed as the ratio of the thermal energy needed to produce a certain quantity of distillate output to the total solar energy input. Mathematically, it is often represented as follows^40^.

$$\eta = (\sum m_{w} \times L)/\sum (I\left( t \right) \times A_{W} \times 3600)$$

${m}_{w}\hspace{0.17em}$= output distillate (kg/m^2^-h), A_w_ = Glass cover area (m^2^), I = Solar radiation incident (W/m^2^), L = Latent heat vaporization (J/kg).

**Figure 11:**
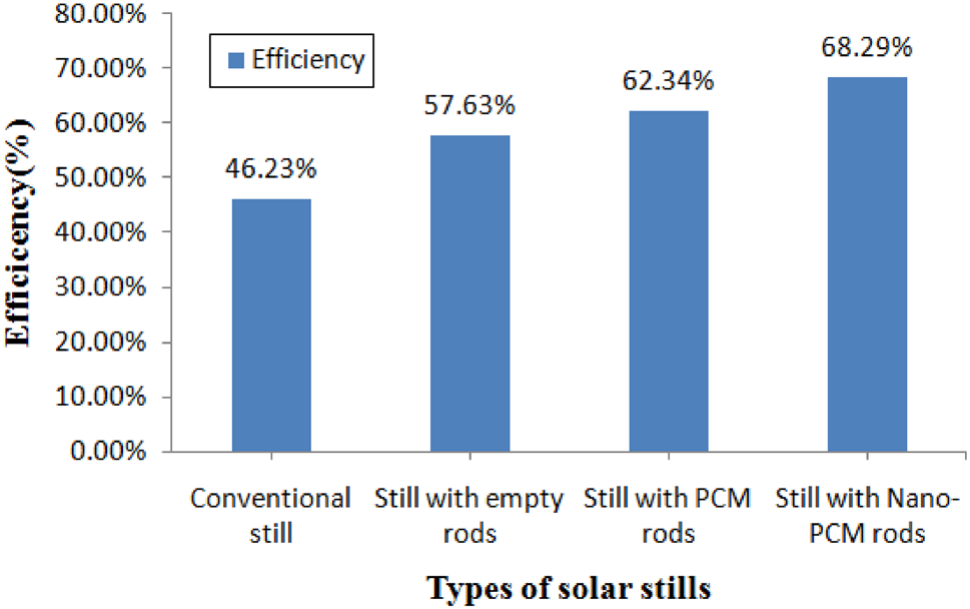
Overall efficiency of different stills.

According to Fig. 11, the efficiency of conventional stills, stills with empty rods, PCM stills, and NPCM stills was 46.23%, 57.63%, 62.34%, and 68.29%, respectively. Hence the overall productivity of the still with an empty rod, the PCM still, and the NPCM, respectively, was 19.12%, 34.31%, and 60.37% higher than that of the conventional solar still.

### Comparison of present study with previous studies

We conducted a comparative analysis between the outcomes of our study and those of similar investigations, as depicted in Table 4, aiming to validate the results of the proposed system. Table 4 reveals that the performance of the elevated single slope solar still with Aluminum oxide-PCM demonstrates significant enhancement compared to other studies, thus supporting our conclusions.

**Table 4:** A comparison between the current findings and previous published studies.

S.no	Type of still	Material	Productivity (%)	Efficiency (%)	References
1	Single slope solar still	Paraffin wax	67.18	–	Kabeel et al. ^41^
2	Single slope solar still	Paraffin wax	28.14	72.63	Dhindsa et al. ^20^
3	Single slope solar still	Paraffin wax with graphite particles	73.8	–	Sharshir et al. ^29^
4	Single slope solar still	Paraffin wax with aluminum oxide particles	60.53	–	Chaichan et al. ^42^
5	Single slope solar still	Paraffin wax	66.7	68	AH Mohammad et al. ^19^
6	Single slope solar still	Paraffin wax with aluminum oxide particles	60.37	68.29	Current study

## Conclusion

In this research work, the improvement in distillate production for the still with nano phase change material rods is contrasted to the stills with empty rods, stills with phase change material rods, and traditional stills. This experiment was carried out to determine whether phase change material at night and nanoparticles during the day solar still will rise the productivity and efficiency. After doing the experiment, it was determined that using nano phase change material significantly enhances yield at the following rate. In readings with empty rods, the temperature of the rod raised to 68 °C at noontime by producing 3176.66 ml of yield from 9:00 am to 5:00 pm.

- Using phase change material in rods has elevated the temperature of the rod at noon time (1:00 pm) to 79 °C while producing 2301.33 ml of produce from 9:00 am to 5:00 pm. Radiation from the sun and yield are higher when there is a lower condensing cover angle, even if production utilizing PCM from 5:00 pm to 9:00 am is 1280 ml.
- Using nano phase change material in rods has elevated the temperature of the rod at noon time (1:00 pm) to 86 °C while producing 2789.66 ml of yield between 9:00 am and 5:00 pm. PCM has a 1487 ml productivity from 5:00 pm to 8:00 am.
- It was discovered that the conventional still, still with an empty rod, PCM still, and NPCM still had respective efficiency values of 46.23%, 57.63%, 62.34%, and 68.29%.
- Compared to traditional still, NPCM still had an increase in productivity and efficiency of 60.37% & 22.06%, respectively.
- The mass concentration of nanoparticles significantly enhances the thermal properties of phase change materials (PCMs). Maintaining an optimal proportion of nano-PCM is crucial for long-term improvements in PCM’s thermo-physical characteristics. This ensures sustained enhancement in specific heat capacity, and latent heat of fusion, contributing to the efficiency and reliability of PCM-based systems over time.
- Using NPCM rises the storage capacity of PCM and reduces charging time, both of which increase productivity at night and solar still efficiency.

### Future scope

Future research exploring alternative geometries like cylinders and pyramids, coupled with modification methods that increase the evaporation surface area, is recommended. Additionally, other nanoparticles such as multi-wall carbon nanotubes, graphite powder, expanded graphite, and erythritol, etc., can also be examined at different weight proportions with phase change materials (PCM) in the solar still. Additionally, experimental energy and exergy analyses will be conducted and assessed based on the experimental data.

## Data Availability

The datasets used and/or analysed during the current study available from the corresponding author on reasonable request.

## Abbreviations

CNT: Carbon nano tubes
EG: Expanded graphite
FGN: Flake graphene nano particles
DSC: Differential scanning calorimeter
FGN: Flake graphene nano particles
GO: Graphene oxide
MWCNT: Multi wall carbon nano tubes
NPCM: Nano phase change material
NTC: Negative temperature coefficient
PCM: Phase change material
SEM: Scanning electron microscope
TEM: Transmission electron microscopy
XRD: X: ray powder diffraction
PW: Paraffin wax
LHS: Latent heat storage
LHF: Latent heat fusion
TES: Thermal energy storage

## List of symbols

Al_2_O_3_: Aluminum oxide
Fe_2_O_3_: Iron oxide
k: Thermal conductivity (W/m K)
Productivity: (Ml/m^2^)
SiC: Silicon carbide
Yield rate: Kg/m^2^
